# Functional transcriptomic analysis of the role of MAB-5/Hox in Q neuroblast migration in *Caenorhabditis elegans*

**DOI:** 10.1186/1471-2164-14-304

**Published:** 2013-05-04

**Authors:** Joel V Tamayo, Mahekta Gujar, Stuart J Macdonald, Erik A Lundquist

**Affiliations:** 1 Department of Molecular Biosciences, Programs in Genetics and Molecular, Cellular, and Developmental Biology, The University of Kansas, 1200 Sunnyside Avenue, Lawrence KS 66045, USA

## Abstract

**Background:**

Directed cell migration is a fundamental process in normal development and in tumor metastasis. In *C. elegans* the MAB-5/Hox transcription factor is a determinant of posterior migration of the Q neuroblast descendants. In this work, *mab-5* transcriptional targets that control Q descendant migration are identified by comparing RNA-seq profiles in wild type and *mab-5* mutant backgrounds.

**Results:**

Transcriptome profiling is a widely-used and potent tool to identify genes involved in developmental and pathological processes, and is most informative when RNA can be isolated from individual cell or tissue types. Cell-specific RNA samples can be difficult to obtain from invertebrate model organisms such as Drosophila and *C. elegans*. Here we test the utility of combining a whole organism RNA-seq approach with *mab-5* loss and gain-of-function mutants and functional validation using RNAi to identify genes regulated by MAB-5 to control Q descendant migration. We identified 22 genes whose expression was controlled by *mab-5* and that controlled Q descendant migration. Genes regulated by *mab-5* were enriched for secreted and transmembrane molecules involved in basement membrane interaction and modification, and some affected Q descendant migration.

**Conclusions:**

Our results indicate that a whole-organism RNA-seq approach, when combined with mutant analysis and functional validation, can be a powerful method to identify genes involved in a specific developmental process, in this case Q descendant posterior migration. These genes could act either autonomously in the Q cells, or non-autonomously in other cells that express MAB-5. The identities of the genes regulated by MAB-5 indicate that MAB-5 acts by modifying interactions with the basement membrane, resulting in posterior versus anterior migration.

## Background

The ability to assay the transcriptional activity of every gene in the genome (transcriptomics) has been facilitated by microarrays and now by next-generation high-throughput transcript sequencing (RNA-seq) [1]. Large-scale transcriptome profiling allows new questions to be asked about the roles of transcription factors in development and the cascades of genes that they influence to drive cell fate and differentiation. Understanding transcriptional programs is also central to understanding disease and pathological conditions resulting from failures in these fundamental processes.

Multicellular organisms present challenges to understanding developmentally-relevant transcriptional programs, as cell-specific differences in gene expression can be masked by expression in multiple cell types. Isolation of specific cell types can solve this problem. In *C. elegans,* embryonic neurons were isolated by fluorescence-activated cell sorting (FACS) and their transcriptional profile assayed by microarrays [2]. In the same report, transcripts from larval neurons were isolated by expression of a tagged poly-A binding protein (FLAG-PAB-1) in neurons and subsequent immunoprecipitation of FLAG-PAB-1 and associated transcripts [2]. Recently, single migrating linker cells of the *C. elegans* male gonad were dissected from living animals and transcriptionally profiled using RNA-seq [3]. Despite these advances, isolation of cell-specific transcripts from model organisms using these techniques is not trivial, and not feasible for every cell type at this time. In the current study we test the efficacy of combining the advantageous genetics and well-understood development of the nematode *C. elegans* with whole organism RNA-seq to identify genes with effects on the migration of the Q neuroblasts. By comparing transcript levels in wild type and various *mab-5* mutant backgrounds defective in Q cell migration, we test if genes with effects on Q migration can be identified by RNA-seq of whole animal lysates.

**Figure 1:**
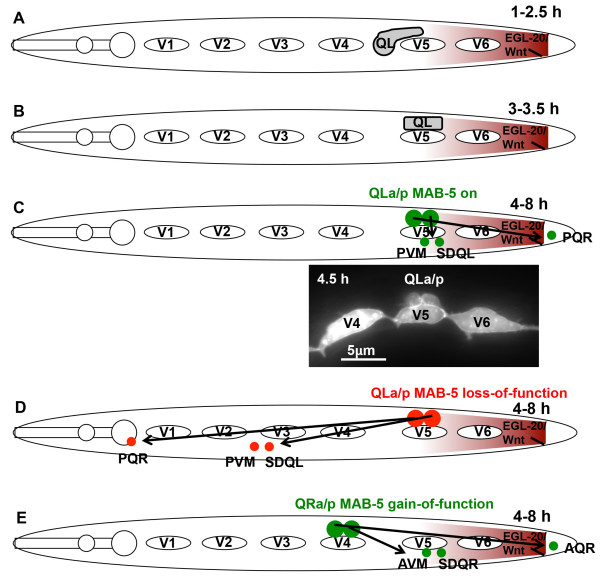
**Q neuroblast migrations in wild type and *mab-5 *mutants. **Schematic diagrams of Q neuroblast migrations are shown. Anterior is to the left. V1-V6 represent the hypodermal seam cells, and the Q neuroblast and its descendants are labeled. The posterior EGL-20/Wnt signal is indicted as a maroon gradient. A-D are diagrams of QL. E is a diagram of QR. **A**) In wild type at 1-2.5 h post hatching, QL protrudes posteriorly over the V5 seam cell. **B**) In wild type at 3-3.5 h post hatching, QL migrates posteriorly and resides on top of the V5 seam cell. **C**) In wild type, QL divides at 4 h post hatching and begins to express *mab-5 *induced by the posterior EGL-20/Wnt signal (indicated by a green cell). Over the next four hours, the QLap descendants undergo a pattern of division and migration resulting in three neurons, PVM, SDQL, and PQR. The diagram is simplified, showing only the final neurons and not the individual divisions and cell deaths that result in the three neurons. The micrograph is of QLap visualized by *lqIs80[scm::gfp]* at 4.5 hours after hatching after QL division and at a time when *mab-5* expression is induced in QLap. Note that V5 has not yet divided, which occurs at approximately 5 h post hatching. **D**) In *mab-5 *loss of function, the absence of *mab-5 *activity (red) results in QLap and descendants migrating anteriorly instead of posteriorly, despite normal initial QL migration posteriorly over V5. PQR migrates anteriorly to the normal position of AQR on the right side. **E**) In *mab-5(e1751)* gain-of-function, *mab-5 *expression in QR (green) results in posterior migration of QRap and descendants despite normal initial anterior migration of QR on top of V4. AQR migrates posteriorly to the normal position of PQR on the left.

**Figure 2:**
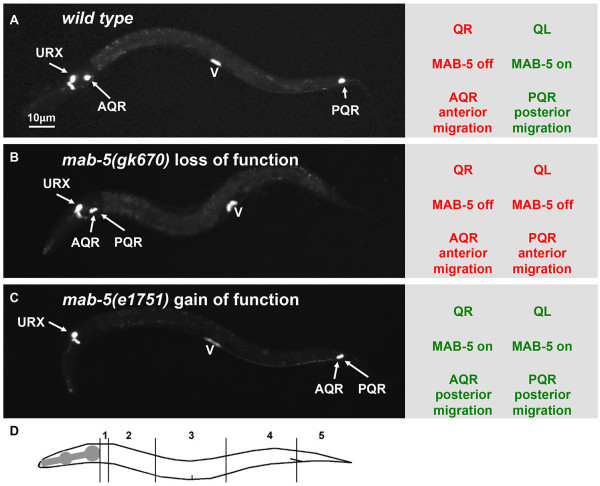
**AQR and PQR migration in wild type and *mab-5 *mutants. **Micrographs of L4 animals expressing *gcy-32::cfp *(*lqIs58)* in AQR, PQR, and the URX neurons are shown. The vulva is marked by expression of *egl-17::gfp *(*ayIs9*). **A**) In wild type, AQR on the right migrates anteriorly to the anterior deirid region, and PQR on the left migrates posteriorly behind the anus in the phasmid ganglion. The diagram to the right indicates the expression status of *mab-5 *AQR and PQR and their precursors. **B**) In *mab-5* loss of function, both AQR and PQR migrate anteriorly. **C**) In *mab-5 *gain of function, both AQR and PQR migrate posteriorly. **D**) Positions along the body used to score AQR and PQR migration defects in mutants in Tables 6 and 7 (see Methods).

The Q cells are neuroblasts that reside bilaterally in the posterior region of the worm (Figure 1A, B, and C). The Q cells, left and right, undergo an identical pattern of division coupled with cell death to produce three neurons [4,5] (Figure 1). While the Q cells divide in a similar fashion, QL descendants migrate posteriorly and QR descendants migrate anteriorly. QL on the left undergoes an initial migration towards the posterior of the worm and divides to give rise to the PVM, SDQL, and PQR neurons [6-8]. These QL descendants continue to migrate posteriorly, with PQR migrating the farthest to reside in the tail of the worm behind the anus (Figure 1A, B, and C Figure 2A). QR on the right undergoes a similar pattern of migration and division, except the cells migrate to the anterior, generating the AVM, SDQR, and AQR neurons [6-8]. AQR migrates the farthest to reside in the head of the worm near the anterior deirid sensory organ (Figure 2A).

Q cell migration occurs in two stages. The initial stage relies on Q cell polarization regulated by the transmembrane molecules UNC-40/DCC, PTP-3/LAR, and MIG-21 [8-10]. When QL undergoes its initial migration towards the posterior, it encounters a graded EGL-20/Wnt signal, which is secreted from muscle and epidermal cells at the posterior of the animal [11-13] (Figure 1A, B, and C). In this second phase of migration, EGL-20/Wnt induces expression of the MAB-5/Hox transcription factor in QL, but not QR, via canonical Wnt signaling (reviewed in [14]) (Figure 1C and Figure 2A).

MAB-5, an Antennapedia-like Hox transcription factor, is essential for the posterior migration of the QL descendants and acts as a determinant for posterior migration of Q descendants [6,13-18]. Loss of function of *mab-5* results in anterior migration of the QL descendants, despite normal initial posterior migration of QL (Figures 1D and 2B). A gain-of-function *mab-5* mutant that results in *mab-5* expression in both QL and QR independent of Wnt signaling causes both QL and QR descendants to migrate posteriorly despite normal initial Q cell migrations (Figures 1E and 2C) [6,18]. Induced autonomous expression of MAB-5 during anterior Q descendant migration results in the cells changing direction to the posterior, and MAB-5 is needed continuously during posterior migration [11,18]. Thus, MAB-5 is both necessary and sufficient for autonomously directing posterior versus anterior migration of Q descendants.

In addition to QL descendants, MAB-5 is expressed in a handful of other cells in the posterior in the mid-L1 stage, including the hypodermal V5 and V6 cells, P7-P12, the M mesoblast, and several posterior body wall muscles [19]. MAB-5 inhibits the expression of the transmembrane molecule MIG-13 in posterior commissural motor neurons generated from P7-P12 [20]. MIG-13 is non-autonomously required for the anterior migration of QR descendants on the right [20]. Thus, MAB-5 might control gene expression outside of the Q cells that non-autonomously controls Q descendant migration. Our whole organism RNA-seq approach has the potential to identify both autonomous and non-autonomous targets of MAB-5 in Q descendant migration. The results reported here do not distinguish between autonomy and non-autonomy, which will be the subject of future study.

It is important to note that QL cell descendants are still generated and still migrate in the absence of MAB-5, but in the opposite direction. Additionally, AQR and PQR are thought to have the same functions of sensing oxygen and regulating social feeding in the worm, even though *mab-5* is expressed in PQR and not in AQR in wild type worms [21]. These factors suggest that MAB-5 is specifically involved in determining the direction of migration of the QL cell descendants and not in the specification of neuronal class identity or function or in their ability to migrate.

MAB-5/Hox represents an excellent opportunity to dissect the transcriptional programs that specify differentiation, in this case posterior versus anterior cell migration. We take advantage of both loss-of-function and gain-of-function alleles of MAB-5 to identify genes whose transcriptional levels are affected by each, and then functionally analyze their roles in Q descendant migrations using RNAi. We find that while some genes are oppositely affected in loss versus gain-of-function backgrounds, the majority of genes are unique in each set, indicating redundancy and combinatorial function of MAB-5 in regulating transcription. We also find that MAB-5 target genes are enriched for those encoding secreted proteins involved in extracellular matrix formation and remodeling, some of which have functional roles in Q descendant migration. This suggests that MAB-5 might alter cell-extracellular matrix interaction to direct posterior migration. Finally, we functionally compare our RNA-seq results with the MAB-5 ChIP-seq results obtained by the ModENCODE project [22] and find that mutant RNA-seq and ChIP-seq are both efficient methods to identify functional targets of MAB-5 in posterior Q descendant migration.

## Results

### Transcriptional profiling of wild-type and *mab-5* mutant L1 animals using RNA-seq

To identify genes whose expression is influenced by MAB-5, we conducted RNA-seq analysis on wild-type worms, two *mab-5* loss-of-function (lof) alleles *e1239* and *gk670* (hereafter, *mab-5(e1239)lof* and *mab-5(gk670)lof*, respectively), and the *mab-5(e1751)* gain-of-function (gof) allele (which we will refer to as *mab-5(e1751)gof*). Total RNA was isolated from each genotype at the early L1 larval stage approximately 4.5-5 h after hatching, after the Q cells had undergone their initial migrations and divisions, and when *mab-5* is activated by *egl-20/Wnt* signaling in QL and QL descendants (see Methods and Figure 1C). RNA from multiple biological replicates of each genotype was then subjected to RNA-seq using Illumina single-end 60-bp reads. Reads were assigned to genes using TopHat [23,24], and pairwise expression differences between genotypes analyzed using the Cuffdiff module in Cufflinks [25-28]. Differential gene expression between genotypes was assessed using an FDR-adjusted *q*-value to account for multiple-testing, and only those genes with significant changes (*q* < 0.05) are discussed. Cuffdiff files for each pairwise comparison can be found in Additional files 1, 2, 3, 4 and 5, and protein-coding genes with significant expression changes can be found in Additional file 6.

Due to our column-based total RNA isolation procedure, and the poly-A selection step prior to RNA-seq, few small, non-coding RNA molecules should be represented in our sequencing data. Nevertheless, some small nuclear snRNAs, small nucleolar snoRNAs, and trans-spliced leader RNAs (*sls* genes) were found to be differentially expressed. It is possible that certain high-abundance genes in these categories are inefficiently removed by poly-A selection. No miRNA genes were identified as differentially expressed, primarily because very few miRNA reads were recovered by our protocol. Given the poor, and potentially inconsistent representation of reads associated small non-coding RNA genes in our study, these genes are excluded from further analysis. Nevertheless, small non-coding RNA genes are included in Additional files 1, 2, 3, 4 and 5, and those showing significant differential expression between genotypes are listed in Additional file 7.

We first determined genes that were down- or upregulated in the two *mab-5* lof backgrounds compared to wild-type. Ten genes were significantly downregulated in *mab-5(e1239)lof* compared to wild-type, while 33 genes were downregulated in *mab-5(gk670)lof* (Table 1 and Additional file 6). Five genes were downregulated in both *lof* mutants, for a total of 38 genes downregulated in at least one comparison. Fewer genes were found to be upregulated in the *lof* backgrounds compared to wild-type: seven in *mab-5(e1239)lof* and six in *mab-5(gk670)lof*, with four in both, for a total of nine genes upregulated in at least one comparison (Table 1 and Additional file 6).

**Table 1:** **Numbers of genes differentially regulated in *mab-5 *loss- and gain-of-function backgrounds**

***mab-5 *loss-of-function compared to wild type**		**Combined**
WT x *e1239 lof *DOWN	10 (5 in WT x *gk670 lof*)	38^1 ^DOWN in LOF combined
WT x *gk670 lof *DOWN	33	(15 UP in GOF combined)
		
WT x *e1269 lof *UP	7 (4 in WT x *gk670 lof*)	9 UP in LOF combined
WT x *gk670 lof *UP	6	(1 DOWN in GOF combined)
***mab-5 *gain-of-function compared to wild type and *mab-5 *loss-of-function**		**Combined**
WT x *e1751 gof *DOWN	41 (10 in *gk670 lof x e1751 gof*)	129^1 ^DOWN in GOF combined
*e1239 lof x e1751 gof *DOWN	17 (10 in *gk670 lof x e1751 gof*)	
*gk670 lof x e1751 gof *DOWN	92 (6 in each)	
		
WT x *e1751 gof *UP	15 (5 in *gk670 lof x e1751 gof*)	82 UP in GOF combined
*e1239 lof x e1751 gof *UP	24 (18 in *gk670 lof x e1751 gof)*	
*gk670 lof *x *e1751 gof *UP	65 (3 in each)	

^1^These categories also included the retired gene R07B7.7, which was not included in further analysis.

We note that the disparity in the number of downregulated genes identified in the comparisons to the two different *lof* mutants is not explained by differences in overall RNA-seq read count for the two mutant genotypes (164 and 166 million reads were collected for *gk670* and *e1239*, respectively). Since *mab-5(e1239)* is a splice donor mutation in the second intron, whereas *mab-5(gk670)* is a deletion of a large region of the locus, it is possible that *mab-5(e1239)* retains some activity and thus more weakly affects gene expression than does *mab-5(gk670)*.

Interestingly, the *mab-5* locus itself was not significantly underrepresented in the two *lof* backgrounds. Indeed, in the *mab-5(e1239)lof* mutant, a 2-bp insertion at *mab-5* was apparent in the RNA-seq reads due to the use of a cryptic 5′ splice site 2 bp downstream from the wild-type site because of an altered splice donor site in the first *mab-5* intron (data not shown) [29]. In the *mab-5(gk670)lof* mutant (Wormbase and E.A.L. unpublished results), the RNA-seq data at *mab-5* was consistent with the known deletion at this locus (data not shown). These data suggest that the *mab-5* transcript might not be subject to nonsense-mediated mRNA decay.

In the *mab-5(e1751)gof* background, 41 genes were downregulated and 15 genes were upregulated compared to wild type, (Table 1 and Additional file 6). We expected that *mab-5* itself would be upregulated in *mab-5(e1751)gof* relative to wild-type, since this mutation causes upregulated *mab-5* expression in multiple cells [19]. While *mab-5* gene expression is higher in the *gof* than wildtype (a 2.8-fold increase, *p* = 0.001) this change is not significant after correcting for multiple tests (*q* = 0.178).

To identify other genes potentially regulated by MAB-5, we compared genes that were differentially expressed between each *lof* mutant and the *gof*, with the idea that this might be a more sensitive and powerful approach than comparing each mutant class separately with wild type (recognizing that this approach does assume loss and gain of *mab-5* expression has opposing effects on expression). Seventeen genes were downregulated in *mab-5(e1751)gof* compared to *mab-5(e1239)lof*, and 92 were down in *mab-5(e1751)gof* compared to *mab-5(gk670)lof*, with 10 in both (Table 1). Six of these genes were also downregulated in *mab-5(e1751)gof* compared to wild-type (Table 1). Twenty four genes were upregulated in *mab-5(e1751)gof* relative to *mab-5(e1239)lof,* and 65 upregulated relative to *mab-5(gk670)lof*, with 18 of the 24 genes differentially expressed in the *mab-5(e1239)lof* comparison also among those in the *mab-5(gk670)lof* comparison.

### Differentially expressed gene sets for *in silico* evaluation and functional testing

Our ultimate goal with this work was to identify and characterize genes arising from our RNA-seq screen that function with MAB-5 in neuronal migration. In this work, we focus solely on those genes that exhibit significant gene expression changes after accounting for multiple testing. This will clearly exclude some true MAB-5 targets, but should provide a gene set with a high probability of success in experimental functional validation.

The *mab-5* gene itself showed significant differential expression only in the comparison between the *mab-5(e1239)lof* and the *mab-5(e1751)gof* (5.0-fold increase in the gof, *q* = 3.4 × 10^–5^). However, the comparisons between the *mab-5(gk670)lof* and *mab-5(e1751)gof* (2.2-fold increase in gof, *p* = 0.0007) and between wild-type and the *mab-5(e1751)gof* (2.8-fold increase in the gof, *p* = 0.001) were nominally significant and in the anticipated direction. This indicates that *bona fide* MAB-5 targets might appear significant in only one of the comparisons of wild-type and the two *lof* backgrounds to the *gof*. Thus, for further characterization we combined genes from all the different comparisons described above into four groups (Table 1): 38 genes total were downregulated in *lof* backgrounds compared to wildtype, while 9 were upregulated. In combined *gof* comparisons to wild-type and the two *lof* backgrounds, 129 genes were downregulated in *gof* and 82 were upregulated.

### Opposing regulation in loss- *versus* gain-of-function

Some transcriptional targets of MAB-5 may be differentially-regulated in opposite directions in the *lof* and *gof* conditions (e.g., a gene downregulated in *mab-5(lof)* might be upregulated in *mab-5(gof)*). Of the 38 genes downregulated in either *lof* mutant compared to wild-type, 15 were upregulated in the *mab-5(e1751)gof* combined gene set (39%), while 1 of the 9 genes upregulated in the *lof* mutants was downregulated in *gof* combined (11%) (Table 2). Thus, there was not a strict concordance between gene expression in the *lof* and *gof* backgrounds, but there was significant overlap. Simple statistical power concerns could explain the lack of precise concordance, but it is also possible that MAB-5 acts redundantly and combinatorially with other transcription factors to influence gene expression.

**Table 2:** **Genes with opposite expression responses to *mab-5***

**Down in lof, UP in gof**	**Up in lof, DOWN in gof**
*C17C3.3*	*F47F2.2*
*C35D10.1*	
*C44B7.6*	
*C50H2.12*	
*C53A3.2*	
*F31F7.1*	
*F56D6.9*	
*C25H3.5/flp-27*	
*K08F8.1*	
*C53A3.2/nitrophenylphosphatase*	
*F42E11.1/p-glycoprotein*	
*C18F10.4/srg-1*	
*T01D3.4/srx-96*	
*T13B5.5*	
*W02B3.6*	

### Genes encoding secreted and transmembrane molecules are enriched among *mab-5-*regulated genes

**Figure 3:**
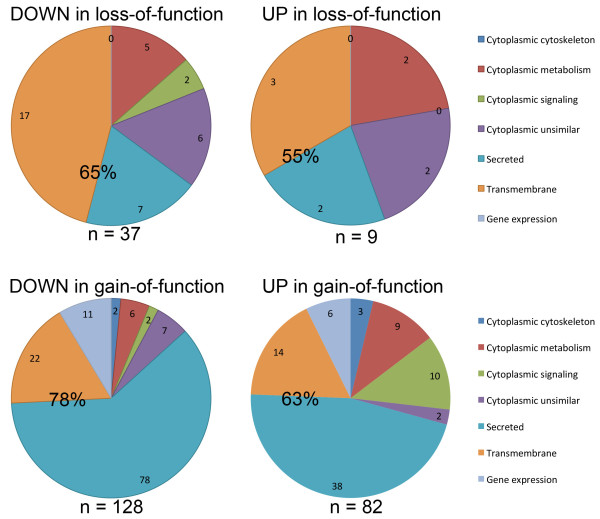
**Genes encoding secreted and transmembrane molecules are enriched among *mab-5 *RNA-seq targets. **The four combined classes of *mab-*5 RNA-seq target genes as described in Results are categorized as to their annotation on Wormbase (see Methods). In each class, the percentages of predicted secreted and transmembrane molecules (teal and orange) are indicated. This enrichment is significant by binomial analysis (Table 3) in each case except UP in loss of function.

For each of the four pools of genes (those up- and downregulated in either *mab-5(lof)* or *mab-5(gof)* backgrounds), we classified genes based on their predicted cellular location and molecular function as annotated in Wormbase (see Additional file 8 and Methods). Within each group, molecules with predicted transmembrane domains and/or a predicted signal sequence (“tmhmm” and “SignalP” annotation on Wormbase) were by far the most abundant class (55-78%, Figure 3). This enrichment for secreted and transmembrane proteins was statistically significant in three of the four groups using a binomial analysis based on the fraction of such genes in the worm genome (Table 3). We note that while the *mab-5(lof)* upregulated gene set did not show a significant excess of tmhmm and SignalP-annotated genes, at 9 genes this is the smallest of the four groups of genes. The remaining genes in the four pooled sets are predicted to encode molecules acting in transcription and gene expression, cytoplasmic signaling, metabolism, and the cytoskeleton (Figure 3).

**Table 3:** **Genes encoding molecules with predicted transmembrane domains and/or a signal peptide are significantly enriched among *mab-5-*regulated genes**

	**Down in lof**	**Up in lof**	**Down in gof**	**Up in gof**	**Total**
Fraction in gene set^1^	24/37	5/9	100/128	52/82	181/256
Binomial p value^2^	p = 0.0368	p = 0.1702	p < 0.0001	p = 0.0129	p < 0.0001

^1^This set includes only protein-encoding genes and not small RNA genes. From gene annotation files from Wormbase, there were 9546 genes with tmhmm and/or SignalP annotation out of 19245 total.

^2^Binomial distribution test comparing fractions of transmembrane and secreted proteins in the gene sets compared to the total fraction in the genome.

### Function and gene ontology of genes affected by *mab-5(lof)*

We used the Database for Annotation, Visualization and Integrated Discovery (DAVID) to perform a Gene Ontology term (GO term) enrichment and functional clustering analysis on the groups of *mab*-*5-*regulated genes (Additional file 9). Genes upregulated in *mab-5(lof)* were enriched for GO terms involving aryl phosphatase and HAD phosphatase activity (2 genes *F44E7.2* and *C53A3.2*). However, genes in this class were few in number and did not return highly significant GO term functional clustering. Genes that were downregulated in *mab-5(lof)* returned GO term enrichment for transmembrane proteins, as expected from the domain analysis above. This group was significantly enriched for GO terms involving secreted glycine-rich proteins (including the three genes *F15E6.8, F15E6.3,* and *F56D6.10*). Terms and a functional cluster involving ATPase activity and nucleotide binding were also identified (Additional file 9). This is due to the downregulation of two genes in *mab-5(lof)*, *pmp-5/T10H9.5* and *pgp-4/F42E11.1*, that encode ABC transporter-family proteins.

### *mab-5(gof)* affects genes involved in extracellular matrix

Among genes upregulated in *mab-5(gof)*, the most significantly enriched domain is related to an extracellular domain encoded by genes induced by fungal infection. Three genes, *fipr-22*, *fipr-23*, and *fipr-24* (Wormbase), harbor this domain (Table 4 and Additional file 9). Two other extracellular domains were also significantly-enriched; the complement C1r/C1s/Uegf/Bmp1 (CUB) domain (three genes), and the thrombospondin type I (TSP1 domain) (three genes). Other enriched domains include aryl phosphatase PHO13/phosphoglycolate phosphatase domain and the HAD-superfamily hydrolase subfamily IIA domain, both of which have been implicated in carbohydrate metabolism. As mentioned above, two genes (*F44E7.2* and *C53A3.2*) harboring these domains were also upregulated in the *mab-5(lof)* background, so their functional relationship to MAB-5 is unclear.

**Table 4:** ***mab-5(gof) *affects secreted and transmembrane proteins associated with the extracellular matrix**

**Up in *mab-5(gof)***		
Fungal-induced	CUB domain	Thrombospondin type I
*C37A5.2/fipr-22*	*C32H11.12/dod-24*	*F14H12.3*
*C37A5.4/fipr-23*	*F35E12.2*	*C37C3.6/mig-6/ppn-1*
*C37A5.8/fipr-24*	*C32H11.13/dct-19*	*F10E7.4/spon-1*
	*F55G11.4*	
Carbohydrate Kinase/phosphatase		
*F44E7.2*		
*F53A3.2/nitrophenylphosphatase*		
*F14B4.2*		
*C50F4.2*		
**Down in *mab-5(gof)***		
Collagen	keratin-like	glycine-rich
*Y42H9B.1/col-115*	*F35A5.4/cys-rich*	*F15E6.8/dct-7*
*F26B1.4/col-58*	*R09B5.5/pqn-54*	*F15E6.3*
*F27C1.8/dpy-5*	*ZK1067.7/abu-14*	*F56D6.10/dct-8*
*C29E4.1/col-90*	*F07H5.8/cys-rich*	
*F38B6.5/col-172*	*AC3.3/abu-1*	Kunitz protease inhibitor
*M195.1/col-77*	*AC3.4/pqn-2*	ZC84.1
*F58F6.1/col-104*	*M02G9.1*	ZK287.4
*F41C6.5/col-173*		F30H5.3
*C46A5.3/col-14*	ShK/metridin	
*F30B5.1/dpy-13*	*F48G7.5*	Histones
*B0491.2/sqt-1*	*F48G7.8*	*F22F1.1/hil-3*
*Y2H9A.2/col-162*	*Y46G5A.29*	*M163.3/his-24*
*C30F2.2/col-187*	*Y45F3A.8*	*Y73B6BL.9/hil-2*
*Y41E3.2/dpy-4*		*F55G1.2/his-59*
Zinc metalloprotease		
*C24F3.3/nas-12*		
*F09E8.6/nas-14*		
*T04G9.2/nas-15*		

This table is a summary of the significant GO terms and functional clusters found in Additional file 9.

Genes downregulated in *mab-5(gof)* were enriched for GO terms and functional clusters involving collagens, secreted metalloproteases and their inhibitors, cysteine-rich repeats found in a variety of secreted proteins, proteins that form disulfide bonds, the ShKT/metridin toxin domain found in secreted proteins, and histones (Table 4 and Additional file 9). This gene group includes: 14 collagen genes; seven genes encoding secreted keratin-like or keratin-associated molecules; three genes encoding secreted astacin metalloproteases (*nas-12,14,15)*; three genes encoding secreted kunitz serine protease inhibitors; four genes encoding secreted proteins containing the ShK/metridin toxin domain (also found in *nas-12,14,15*); and four histone genes. Three genes encoding secreted glycine-rich repeats were downregulated in *mab-5(gof)*, but these were also downregulated in *mab-5(lof)*, suggesting that they might not be relevant targets.

In sum, this GO term enrichment analysis suggests that MAB-5 predominantly regulates genes encoding secreted and transmembrane molecules. Based on the *mab-5(gof)* results, genes upregulated by MAB-5 tend to encode secreted and transmembrane molecules with domains involved in signaling, cell adhesion, and fungal defense (CUB, TSP1, and FIPR domains). Genes downregulated by MAB-5 tend to encode secreted molecules that are involved in the formation and remodeling of the extracellular matrix (collagens, and secreted metalloproteases and protease inhibitors), suggesting MAB-5 might alter cell interactions with the extracellular matrix. Indeed, two genes upregulated by *mab-5(gof)*, *spon-1/F-spondin* and *mig-6/Papilin*, encode secreted molecules containing both kunitz-type proteinase inhibitor domains and TSP1 domains, and are important for cell migration [30,31].

### Comparison of MAB-5 RNA-seq data with modENCODE ChIP-seq data

Regions of the genome with which MAB-5 interacts physically were identified previously by chromatin immunoprecipitation and DNA sequencing (ChIP-seq) as part of the ModENCODE project [22]. We compared a list of potential MAB-5 ChIP seq targets (Additional file 5 in [22]) with our RNA seq data set (Table 5): 2/37 genes downregulated in *mab-5(lof)* were present in the annotated ChIP seq targets, and 1/9 *mab-5(lof)* upregulated genes were included. In the *mab-5(gof)* comparisons, 4/128 downregulated genes and 8/82 upregulated genes were included. Thus, while some RNA-seq and ChiP-seq targets were in common, there was not strict concordance between targets identified by the two methodologies. One caveat is that the ChIP-seq data were gathered from L3 larval animals whereas our RNA-seq data were gathered from L1 larval animals. It is also possible that MAB-5 regulates many genes indirectly, or that MAB-5 is redundant with other transcription factors in the regulation of many genes.

**Table 5:** MAB-5 ChIP seq targets are among MAB-5 RNA seq targets

**DOWN in lof**	**UP in lof**	**Down in gof**	**UP in gof**
*K08F8.1*	*F46C5.1*	*ZK180.5*	*K08F8.1*
*F15E6.3*		*F15E6.3*	*F08B1.1/vhp-1*
		*C04F5.7ugt-63*	*F46C5.1*
		*M163.3/his-24*	*C37C3.6/mig-6*
			*Y38E10A.13*
			*C50F7.5*
			*F26D12.1/fkh-7*
			*C08C3.1/mab-5*

The *mab-5* gene itself was one of the 15 genes included in both the RNA-seq and ChIP-seq data sets. *mab-5* has been shown to regulate its own expression by a positive feedback mechanism to maintain *mab-5* expression in the Q neuroblast descendants during their posterior migrations [11,18]. Also identified by both methods was *K08F8.1*, which encodes a MAP kinase activated protein kinase similar to vertebrate MAPKAP3. *K08F8.1* also showed opposite regulation in *mab-5 lof* and *gof* backgrounds (K08F8.1 was downregulated in *lof* and upregulated in *gof;* Table 2).

### Posterior AQR migration in *mab-5(e1751)gof* as a sensitized background to identify *mab-5* targets that function in posterior migration

The goal of this work is to identify genes regulated by MAB-5 that function in Q descendant migration. MAB-5 is required for posterior migration of the Q neuroblast descendants, and can also drive posterior migration of the QR descendants, which normally migrate anteriorly (e.g. *mab-5(e1751)gof* results in posterior migration of QR descendants including AQR; Figure 2C). Genes upregulated in *mab-5(e1751)gof* and downregulated in *mab-5(lof)* are good candidates for those that might mediate posterior Q descendant migration downstream of MAB-5. Loss of such a gene’s function might modify *mab-5(e1751)gof* posterior AQR migration, resulting in more anterior AQR positioning. Since AQR normally migrates anteriorly, posteriorly-migrating AQR in *mab-5(e1751)gof* might represent a sensitized background to identify effects on posterior migration that might be missed in posterior PQR migration. Thus, we tested genes identified by our RNA-seq analysis for their ability to modify posterior migration of AQR in the *mab-5(e1751)gof* mutant.

### *ceh-20* and *unc-62* are epistatic to *mab-5(gof)* in posterior AQR migration

As proof of principle of using posterior AQR migration in *mab-5(e1751)gof* as a background to define *mab-5* targets that affect Q cell descendant migrations, we studied genetic interactions of *mab-5(e1751)gof* with two genes previously shown to interact with the Hox gene *lin-39* in Q descendant migration, *ceh-20* and *unc-62*[32]. *ceh-20* encodes a homeodomain transcription factor of the Extradenticle/Pbx family, and *unc-62* encodes a homeodomain transcription factor of the Homothorax/Meis1 family [33], vertebrate counterparts of which can act as cofactors with other Hox factors to regulate gene expression [34].

Both *ceh-20* and *unc-62* controlled anterior-posterior migrations of the Q neuroblasts descendants, including AQR and PQR (Table 6). In *ceh-20* and *unc-62* mutants, a fraction of AQR and PQR neurons migrated in the wrong direction. *unc-62* and *ceh-20* are both predicted MAB-5 targets by ChIP-seq [22], suggesting that they might be direct transcriptional targets of MAB-5. Nevertheless, neither gene was identified in the RNA-seq analysis presented here. We speculate that this is due to *mab-5-*independent expression, as *ceh-20* broadly expressed in many cells including those that express *mab-5* and those that do not [32].

**Table 6:** ***ceh-20 *and *unc-62 *control AQR and PQR migration and modify *mab-5(e1751)gof***

	**AQR position (%)**^**1**^						**PQR position (%)**^**1**^					
**Genotype/strain**	**1**	**2**	**3**	**4**	**5**	**n**	**1**	**2**	**3**	**4**	**5**	**n**
*wild-type*	100	0	0	0	0	100	0	0	0	0	100	100
*mab-5(e1751)*	0	0	0	8	92	100	0	0	0	0	100	100
*ceh-20(ay9)*	100	0	0	0	0	100	6	16	31	22	25	100
*ceh-20(mu290)*	22	50	25	3	0	32	5	3	10	46	36	39
*ceh-20(RNAi)*	89	7	1	3	0	100	0	0	9	10	81	100
*unc-62(mu232)*	56	34	8	2	0	106	9	38	20	19	14	105
*unc-62(e917)*	88	0	0	0	12	25	33	0	0	0	67	32
*unc-62(e644)*	51	36	8	0	5	39	26	11	9	0	57	35
*ceh-20(RNAi); mab-5(e1751)*	0	0	6	35	59^2^	100	0	0	4	6	90	200
*unc-62(mu232); mab-5(e1751)*	0	2	12	51	35^2^	100	0	1	2	15	82	100
*unc-62(e644); mab-5(e1751)*	36	10	5	5	44^2^	51	17	4	2	2	75	51
*unc-62(e917); mab-5(e1751)*	43	4	0	8	45^2^	28	0	0	0	0	100	28

^1^AQR and PQR position as described in Methods and Figure 2D.

^2^Significantly different (p < 0.001) compared to mab-5(e1751) alone (Fisher’s Exact Test).

We tested if *ceh-20* and *unc-62* loss of function modified the *mab-5(e1751)* gain-of-function phenotype using a combination of mutations and RNAi. Indeed, in *mab-5(e1751); unc-62* and *mab-5(e1751); ceh-20* double mutants, significantly fewer AQR neurons migrated posteriorly than in *mab-5(e1751)gof* alone (Table 6). Thus, genes that interact with *mab-5* can modify the *mab-5(e1751)gof* posterior AQR phenotype, providing proof of principle that this screen can identify genes that functionally interact with *mab-5* in posterior migration. It is possible that *unc-62* and *ceh-20* act downstream of *mab-5* as suggested by ChIP seq, but it is also possible that they act together with *mab-5*. Indeed, MAB-5 and CEH-20 act together to control transcription of the *egl-1* gene to initiate programmed cell death in the Q lineage [35].

### RNA-seq targets functionally interact with *mab-5(gof)* in posterior AQR migration

We tested *mab-5* RNA-seq targets for function in Q descendant migration using RNAi by feeding (see Methods) [36]. Based on availability in the RNAi feeding library (Source Bioscience, Nottingham, UK), we tested RNAi of 57 genes upregulated in *mab-5(gof)*, and 23 genes downregulated in *mab-5(lof)* for the ability to modify posterior AQR migration in *mab-5(e1751)gof* (Additional file 10). Genes were tested in groups, and each independent experimental grouping included *ceh-20* RNAi as a positive control to ensure RNAi efficacy. Each gene was retested at least once to ensure consistency of results.

*mab-5(e1751)gof* caused 92% of AQR neurons to migrate posteriorly behind the anus to the normal posterior position of PQR, with the remaining 8% just anterior to this position (Table 7 and Figure 1). *ceh-20(RNAi)* reduced posterior AQR in *mab-5(e1751)gof* to 59% (p < 0.001) (Tables 6 and 7). We tested three gene classes by RNAi: those upregulated in *mab-5gof (*49 genes), those downregulated in *mab-5lof* (15 genes), and those in both (eight genes), for a total of 72 genes tested. Sixteen of the 49 *mab-5(gof)*-upregulated genes, 3/15 *mab-5lof* genes, and 3/8 genes in both showed significantly fewer AQR in the posterior compared to the *mab-5(e1751)gof* alone (p < 0.05) (Table 7). RNAi of the remaining 50 genes had no effect on *mab-5(e1751)gof* (data not shown).

**Table 7:** RNAi of RNA-seq and ChIP-seq candidates

	**AQR position (%) (n ≥ 100; p ≤ 0.05 compared to *mab-5(e1751*)**					
**Genotype**	1	2	3	4	5	
*wild-type*	100	0	0	0	0	
*mab-5(e1751)*	0	0	0	8	92	
*ceh-20(RNAi)*	89	7	1	3	0	
*mab-5(e1751); ceh-20(RNAi)*	0	0	6	35	59	
**RNAi in a *mab-5*( *e1751 *) background**						
**Up in *mab-5*( *gof *)**						Description
**Secreted/transmembrane**						
*C37C3.6/mig-6*^*3*^	0	0	2	24	74	*Papilin protease inhibitor*
*F16C3.2*	0	0	1	21	74	Secreted
*K02G10.7/aqp-8*	0	0	0	26	74	*aqp-8* aqueporin
*F17E9.11/lys-10*	0	0	0	23	77	Lysosomal protein
*C35D10.1*^*1*^	0	0	0	22	78	Transmembrane DUF2012
*C02A12.4/lys-7*	0	0	1	20	79	Lysosomal protein
*F11C7.5/osm-11*	0	0	0	21	79	Secreted
*F10E7.4/spon-1*	0	0	0	20	80	F-spondin
*C37A5.4/fipr-23*	0	0	1	16	83	Fungal-induced, secreted
**Gene expression**						
*C08C3.2/bath-15*	0	0	0	28	67	*BTB-MATH*
*C08C3.3/mab-5*^*2*^	0	0	1	22	77	Hox
*F26D12.1/fkh-7*^*3*^	0	0	1	18	81	Forkhead
**Cytoplasmic signaling/cytoskeleton**						
*K08F8.1*^*1,2*^	0	0	1	24	75	MAPKAP3-like
*T04H1.9/tbb-6*	0	0	0	21	76	Beta tubulin
*T07C4.9/nex-2*	0	0	0	23	77	Annexin
*Y17G7B.14/arrd-8*	0	0	0	20	80	Arrestin
*F59A1.9*	0	0	0	20	80	F-box containing
*M60.7*	0	0	1	17	82	Ankyrin-repeat containing
**Metabolism**						
*C53A3.2*^*1*^	0	0	0	25	75	Nitrophenylphosphatase
**Down in *mab-5*( *lof *)**						
*ZC443.6/ugt-16*	0	0	0	22	78	UDP Glucuronosyltransferase
*W10G11.1*	0	0	1	18	81	Secreted cys-rich DUF19
*F48C1.8*	0	0	0	19	81	Secreted, conserved
**ChIP seq targets**						
*T23G4.2*	0	0	0	38	62	Unsimilar cytoplasmic
*T24D1.4/tag-179*	0	0	0	33	67	Transmembrane glycosyltransferase ALG10
*C55B7.7/gly-2*	0	0	0	33	67	Secreted glycosyltransferase, family 18
*T23G4.1/tlp-1*	0	0	0	30	70	Zinc-finger transcription factor
*F22D6.2*	0	0	0	29	71	AN1-like zinc finger
*F54F2.2/zfp-1*	0	0	0	27	73	Zinc finger PHD-like
*F32E10.1/nol-10*	0	0	0	26	74	Nucleolar protein
*M05B5.4*	0	0	1	23	76	Phospholipase A2-like
*R13A5.5/ceh-13*	0	0	0	24	76	Hox-B1/Labial
*W09G12.8*	0	0	0	24	76	Peptide N-glycanase/PAW domain
*F33G12.6*	0	0	0	24	76	Protein phosphatase 2C-like
*R01E6.3/cah-4*	0	0	0	24	78	Carbonic anhydrase
*B0361.2*	0	0	0	21	79	CwfJ C-terminal domains
*F42C5.9*	0	0	0	20	80	Actin-like PH domain
*F21A10.2*	0	0	0	20	81	Transmembrane myelin regulatory factor
*DY3.2/lmn-1*	0	0	0	19	81	Nuclear lamin
*C09H6.3/mau-2*	0	0	0	19	81	Chromatid cohesion factor

^1^Also DOWN in mab-5(lof).

^2^Also a MAB-5 top 50 ChIP seq target.

^3^Also a MAB-5 ChIP seq target.

^4^AQR position as described in Methods and Figure 2D.

As expected, RNAi of *mab-5/C08C3.2* itself, part of the upregulated in *mab-5(gof)* category, significantly reduced posterior AQR migration (Table 7). Two additional genes confirmed by RNAi have previously been implicated in controlling cell migration, *mig-6/papilin* and *spon-1/F-spondin*[30,31], validating our genomics approach to identifying functional targets of MAB-5*.* In addition, four of our RNAi hits are predicted MAB-5 ChIP-seq targets, *mab-5/C08C3.3* itself*, fkh-7/F26D12.1*, *mig-6/C37C3.6/Papilin,* and *K08F8.1*.

None of these RNAi treatments affected PQR migration in *mab-5(e1751)gof*, nor did they cause AQR or PQR migration defects in a wild-type background (data not shown). This is surprising for *mab-5*, since mutations in this gene typically cause nearly all PQR to migrate anteriorly. This could be due to the low efficacy of RNAi and the possible insensitivity of the *mab-5* locus to RNAi. Our data suggest that posterior AQR migration in *mab-5(e1751)gof* is a sensitized system to detect effects on cell migration that might be masked in anterior AQR or posterior PQR migration because of genetic redundancy.

### ChIP-seq targets functionally interact with *mab-5(gof)*

We tested the top 50 ModENCODE ChIP-seq targets [22] predicted to encode proteins and that were available in the RNAi library for interaction with *mab-5(e1751)gof* AQR migration (Table 7 and Additional file 8). RNAi of 19 of these 50 significantly reduced posterior AQR migration in *mab-5(e1751)gof* (p < 0.05)*.* Two of these 19 were also in the upregulated in *mab-5(e1751)gof* set (*K08F8.1* and *mab-5* itself). None affected AQR or PQR migration alone, nor did any affect PQR migration in *mab-5(e1751)gof* (data not shown). We note that *unc-62* is among the top 50 ChIP-seq targets, but *unc-62* RNAi did not modify *mab-5(e1751)gof* posterior AQR migration as did *unc-62* mutants (Table 6). This is likely due to inefficient RNAi of the *unc-62* locus and indicates that our RNAi studies are likely not identifying all of the genes that modify *mab-5(e1751)gof* posterior AQR migration.

In total, 72 genes identified by RNA-seq (up in *gof* plus down in *lof*, with eight in both) were tested by RNAi, and 22 affected AQR migration in *mab-5(e1751)gof* (31%). Fifty ChIP-seq targets were tested, and 19 had an effect (38%). Thus, both methods (ChIP-seq and RNA-seq) were similarly efficacious in identifying potential functional targets of MAB-5 in posterior Q descendant migration.

## Discussion

Cell-specific transcriptome analysis in multicellular organisms usually relies on isolating RNA samples from isolated single cells or cell populations. In invertebrate model organisms such as *C. elegans*, isolation of RNA from specific cell populations can be challenging, but has been achieved through cell sorting, cell-specific expression of an immuno-tagged poly-A binding protein and subsequent immunoprecipitation of mRNAs [2], and single cell dissection [3]. While powerful, these techniques are not applicable to all cell types at this time. In this work, we use whole organism RNA-seq combined with mutant analysis in *C. elegans* to identify genes involved in Q neuroblast migration. The MAB-5/Hox transcription factor is necessary and sufficient for posterior Q descendant migration and likely controls the expression of genes that direct posterior versus anterior migration. Our idea is that by identifying transcripts in staged L1 animals whose expression changes in *mab-5* loss and gain of function mutants, the problems with using whole animal RNA-seq to identify genes involved in Q migration (e.g. expression in many or all cell types) might be mitigated. We then used RNAi and the functional test of modifying posterior AQR migration in *mab-5(e1751)gof* to validate our approach. This approach will not distinguish between genes that act autonomously in the Q cells or non-autonomously, as *mab-5* can act both autonomously and non-autonomously in Q descendant migration. Further downstream experimentation will be required to distinguish these possibilities.

### Mutant RNA-seq revealed genes regulated by *mab-5* that function in Q descendant migration

We conducted RNA-seq on staged L1 animals at a time when QL has just divided and *mab-5* expression was activated in the Q cells by EGL-20/Wnt. By staging animals in this manner, temporal variability in gene expression was minimized. For example, a gene might be expressed in many cells in embryogenesis but in fewer cells at the L1 time point. To focus on genes that might be important in Q descendant migration, we compared RNA-seq profiles from wild-type, two loss-of-function *mab-5* mutants, and a gain-of-function *mab-5* mutant. We focused on genes that were upregulated in the *mab-5gof* and genes that were downregulated in *mab-5lof* as the best candidates for *mab-5* targets that drive posterior Q migration. RNAi was used to test if these genes were required for posterior AQR migration in the *mab-5(e1751)gof* mutant, which is a sensitized background for posterior migration. In this manner we identified 22 genes that modified posterior AQR in *mab-5(e1751)gof* when subject to RNAi. These genes include known players in cell migration (e.g. *spon-1/F-spondin* and *mig-6/papilin)* as well as genes with no previously known role in cell migration. *mab-5* is expressed in a handful of cells in the posterior in addition to QL. Our studies do not address autonomy of function of genes in QL, and these genes might act autonomously with *mab-5* in QL, or might non-autonomously affect Q migrations along with *mab-5* in other *mab-5*-expressing cells in the posterior. In any event, whole animal RNA-seq combined with mutational analysis and functional validation succeeded in identifying *mab-5* transcriptional targets involved in Q migrations.

We envision caveats with our whole-organism mutant RNA-seq approach limiting its effectiveness in identifying particular subsets of the relevant genes. Our approach should readily identify genes expressed only in the Q cells whose expression is dependent on *mab-5*, as well as *mab-5*-dependent genes expressed in multiple cells, assuming the change in expression is sufficient to be detectable in our study. More difficult to identify by our approach are genes expressed in multiple cells whose expression is dependent upon *mab-5* in only a subset of cells. This is the case with the known *mab-5* transcriptional target *mig-13*[20]. MAB-5 inhibits expression of the transmembrane molecule MIG-13 in posterior commissural motor neurons generated from P7-P12 [20]. *mig-13* is also expressed in cells independently of *mab-5*, including the intestinal valve cells and anterior commissural motor neurons [20]*.* In our RNA-seq results, *mig-13* expression was not significantly altered (after correction for multiple tests) in any comparison. This was likely due to expression of *mig-13* in cells not controlled by *mab-5* increasing background noise, preventing us from detecting the expected MAB-5-dependent expression change in this bona fide *mab-5* target*.*

Further functional validation of the candidate genes coming out of our RNA-seq study may yield additional relevant loci, since *mig-13* expression was consistently reduced in the *mab-5(e1751)gof* genotype in comparison to wild-type and both loss-of-function mutants (Additional file 11), consistent with *mab-5* repression of *mig-13*[20]. In addition, in comparison with wild-type, both loss-off-function alleles had increased *mig-13* expression as expected. The observed expression changes in these five comparisons were small, and only two were nominally significant (*p* < 0.05). Functional validation of genes showing the *mig-13* pattern might identify more relevant candidates, even if the changes are slight and not formally significant. However, the success rate would likely be lower given the expected increase in false positives when considering genes that did not pass a stringent statistical threshold controlling for multiple tests.

The use of posterior AQR migration in *mab-5(e1751)gof* as a sensitized background was illustrated, as none of the genes we tested by RNAi had effects on PQR or AQR migration in a wild-type background but did reduce posterior AQR migration in *mab-5(e1751)gof*. This could in part be due to the variable efficacy of RNAi as evidenced by *mab-5* RNAi having no effect on PQR migration in wild type. It is also likely that transcriptional cascades downstream of *mab-5* involve redundancy of function that is unmasked in the *mab-5(e1751)gof* background.

In sum, 22 genes were identified that were regulated by *mab-5* in our expression study, and modified posterior AQR migration in *mab-5(e1751)gof* via RNAi. Nineteen were upregulated in *mab-5gof*, six were downregulated in *mab-5lof* and three genes showed both patterns (*C35D10.1*, *C53A3.2*, and *K08F8.1*) (see Additional file 10). These three genes showing opposing patterns of expression in the *mab-5* loss- and gain-of-function genotypes are excellent candidates for targets of *mab-5* that control Q migrations. *C35D10.1* encodes a conserved transmembrane protein of the DUF2012 family, *K08F8.1* encodes a conserved serine/threonine kinase of the MAPKAP3 family, and *C53A3.2* encodes a potential nitrophenylphosphatase that regulates phosphoglycolate metabolism [37]. In addition, *K08F8.1* is a ChIP-seq target of MAB-5 (see Additional file 10) [22], making it the only gene in this analysis that satisfies each of the criteria described here (opposite transcriptional response to *mab-5* activity, functional modification of posterior AQR migration in *mab-5(e1751)gof*, and a MAB-5 ChIP-seq target).

### *mab-5* targets are enriched for genes encoding molecules involved in extracellular matrix

Overall, a large fraction of the genes we identified as differentially-expressed in response to MAB-5 encoded secreted and transmembrane proteins (Figure 3 and Table 3). Of these, GO terms for genes involved in extracellular matrix formation and rearrangement were enriched (Table 4). Genes upregulated by *mab-5(gof)* were enriched for those encoding CUB domains and thrombospondin type I domains. Those downregulated by *mab-5(e1751)gof* were enriched for genes encoding collagens, zinc metalloproteases, and Kunitz protease inhibitors, which form and modify extracellular matrices. Genes encoding the ShK/metridin domain, found in sea anemone toxins that are inhibitors of voltage gated potassium channels [38], were enriched, as well as a glycine-rich domain of unknown function.

Relatively fewer genes were found that encode molecules involved in gene expression, such as transcription factors, although the *bath-15,* a potential transcriptional regulator, and *fhk-7*, a forkhead family transcription factor, both modified *mab-5(gof)* posterior AQR. *mab-5* predominantly affected genes encoding secreted and transmembrane molecules, suggesting that *mab-5* influences cell-extracellular matrix interactions. This is consistent with the *mab-5* phenotype in QL descendants where only direction of migration is affected but not cell division, cell death, or cell fate.

Migrating cells must modify interactions with basements membranes, including remodeling during normal development as well as invasion across basement membranes in normal development and tumor metastasis (e.g. [39-41]. In *C. elegans*, the gonadal anchor cell crosses a basement membrane to invade the vulval epithelium, reminiscent of metastatic cells, a process regulated by Fos, Daughterless, and Evi-1 transcription factors [42-45]. Furthermore, precise basement membrane gapping, sliding, and targeted adhesion remodels tissue boundaries during attachment of the vulval epithelium to the uterine cells [46]. The Q descendants migrate between the hypodermis and basement membrane, and therefore must navigate through or disrupt hypodermal attachments to the basement membrane. MAB-5/Hox might regulate basement membrane reorganization that results in posterior migration.

Two genes previously implicated in cell migration, *spon-1/F-spondin* and *mig-6/papilin,* were upregulated in *mab-5gof* and modified posterior AQR of *mab-5gof* when subject to RNAi. *spon-1::gfp* and *mig-6::gfp* are expressed broadly in body wall muscles and other cells in embryos and larvae [30,31]. That these two broadly expressed genes were identified as *mab-5* targets by whole animal RNA-seq indicates the power of combining next-generation RNA sequencing with mutant analysis (i.e. the increase in expression of these genes in *mab-5(e1751)gof* could be detected despite broad background expression). Expression of *spon-1* and *mig-6* in the Q cells has not been demonstrated, but different MIG-6 isoforms can act autonomously and non-autonomously in gonadal distal tip cell migration [30], indicating that a secreted molecule can act autonomously on the cell producing it. Future studies will be aimed at determining the autonomy of function of *mab-5* targets and their mechanistic roles in driving posterior migration of Q descendants in response to the MAB-5/Hox transcription factor.

## Conclusions

We used whole organism RNA seq combined with mutant analysis to identify genes regulated by the MAB-5/Hox transcription factor in Q neuroblast descendant anterior-posterior migrations in *C. elegans*. Wild-type, *mab-5* loss-of-function and *mab-5* gain-of-function strains were subjected to RNA seq, and genes upregulated or downregulated in the different mutant lines were identified. Genes regulated by MAB-5 were enriched for those encoding secreted and transmembrane molecules that affect extracellular matrix, indicating that MAB-5 might mediate cell interactions with the extracellular matrix. 31% of genes upregulated in the *mab-5* gain-of-function and downregulated in *mab-5* loss-of-function had an effect on Q descendant migration when perturbed by RNAi, similar to the 38% of genes identified previously by ChIP seq. Our results indicate that whole organism RNA seq combined with mutant analysis can identify genes involved in a specific developmental process, in this case Q neuroblast descendant anterior-posterior migration.

## Methods

### Genetics

*C. elegans* were cultured by standard techniques and reared at 20°C. The following strains were subject to mRNA isolation and RNA-seq: wild-type *mab-5* LE2544 (*lqIs80 IV; lqIs58 V*); Loss of function *mab-5* LE2962 (*mab-5(e1239) III; lqIs80 IV; lqIs58 V*) and LE2963 (*mab-5(gk670) III; lqIs80 IV; lqIs58 V*); Gain of function *mab-5* LE3037 (*mab-5(e1751) III; lqIs80 IV; lqIs58 V*). The following strains were also used: LE1940 (*lqIs58* V), LE2959 (*mab-5(e1751) III; IlqIs58 V*), LE3372 (*ceh-20(ay9) III; lqIs58 V*), LE3391 (*unc-62(mu232) V; lqIs40 I*), LE3397 (*unc-62(mu232) V; mab-5(e1751) III; lqIs40 I*), LE3272 (*unc-62(e644) V; lqIs40 I*), LE3402 (*unc-62(e644) V; mab-5(e1751) III; lqIs40 I*), LE3389 (*unc-62(e917) V; lqIs40 I*), LE3399 (*unc-62(e917) V; mab-5(e1751) III; lqIs40 I.* The *lqIs40* and *lqIs58* transgenes consist of *Pgcy-32::cfp*, and the *lqIs80* transgene consists of *Pscm::gfp*[6]. The strain NH2842 containing the *ayIs9[Pegl-17::gfp]* transgene was also used [47].

### L1 larval staging

Animals of each genotype were grown on 25 nematode growth medium (NGM) plates (10 cm) until they had nearly depleted the bacterial food source (HB101 *E. coli*), and when a majority of the animals were gravid adults. Embryos were separated from gravid adults by standard bleach treatment, placed on ten NGM plates (5 cm) seeded with OP50 *E. coli*, and allowed to hatch and develop as L1 larvae for 18 h at 20°C. Starting at 18 h post plating, larvae were inspected for the position of the Q neuroblasts visualized by the *lqIs80[Pscm::gfp]* transgene included in each strain. When a majority of the larvae displayed Q neuroblasts that were migrating, or that had migrated above their respective seam cells but had not yet divided (~3-3.5 h post-hatching; see Figure 1A and B), the larvae were washed from the plates, washed 3 times in M9 buffer to remove bacteria, and allowed to sit for 1 h at 20°C to clear remaining bacteria in the gut. The animals were inspected again by microscopy to ensure that they were at the stage when *mab-5* is activated in QL by *egl-20/Wnt* (i.e., a majority of the Q cells had migrated and divided, approximately 4-5 h post hatching (see Figure 1C). By this method, between 70% and 90% of the larvae were at the desired stage, depending on genotype, with some slightly younger or older. A small percentage (3-5%) were significantly older than desired, possibly because of slight egg laying variation within and between strains. Larvae were pelleted by centrifugation, and most of the M9 buffer was removed, leaving approximately 200 μl. Larvae were flash frozen in liquid nitrogen and subjected to total RNA isolation. For each genotype, three independent preparations were isolated for biological replication except for the *mab-5* wild-type strain wild-type LE2544, which had four independent isolations.

### RNA isolation

To 200 μl of larvae in M9 buffer [48], 200 μl of 2× Proteinase K lysis buffer (200 mM Tris pH 8.0, 2 mM EDTA, 400 mM Sodium Acetate, and 1% SDS) were added. Proteinase K was added to a final concentration of 400 μg/μl, and the mixture was incubated at 65°C with occasional gentle mixing. This acute Proteinase K treatment led to complete digestion of larvae after 10 minutes. The preparation was extracted with 48 phenol::48 chloroform::2 isoamyl alcohol (3×) and 98 chloroform::2 isoamyl alcohol (2×). RNA was precipitated using 0.5 volumes of 100% ethanol and centrifugation. The pellet was washed with RNase-free 70% ethanol, and resuspended in 60 μl of RNase-free ddH_2_0. The RNA was further purified using RNeasy spin columns (Qiagen) following manufacturer protocols. Small RNAs such as microRNAs, snRNAs, and snoRNAs are not consistently retained in the samples after RNeasy treatment.

Total RNA yield was quantified using a Nanodrop spectrophotometer, and RNA quality was ensured using an Agilent Bioanalyzer as well as agarose gel electrophoresis. Thirty micrograms of total RNA at 1 μg/μl was sent to Cofactor Genomics (St. Louis, MO) for poly-A purification, library construction, and sequencing.

### RNA-seq data collection

Each of the 13 independent L1 total RNA samples (four for wildtype strain LE2544, and three for each of the mutant strains LE2962, LE2963, and LE3037) were used to generate an unpaired 60-bp Illumina mRNA library, and each library was run over two lanes of an Illumina GAIIx sequencer. Biological and technical replicates were rationally arranged over flow cells and lanes in order to account for any lane-to-lane variation in read count or sequencing quality. Approximately 31 million reads were collected per lane, for a project total of 0.8 billion raw reads. Raw reads have been deposited in the Sequence Read Archive (SRA) under project accession number SRP017621.

### Gene expression analysis

A pre-built sequence index, and associated NCBI-format annotation file, of the reference *C. elegans* genome was downloaded from http://tophat.cbcb.umd.edu/igenomes.html. Raw reads were aligned to this reference using TopHat version 1.3.1 [23,24] with default parameters, except that the “no-novel-juncs” flag was used to search only those currently annotated exon-exon junctions. Reads from replicate lanes were pooled, yielding one TopHat assembly for each biological sample. On average 85% of the raw reads aligned to the genome. Expression differences among genotypes were generated for all pairwise comparisons using Cuffdiff, part of the Cufflinks package, version 1.0.3 [25-28]. Default running parameters were used, with the addition of the “frag-bias-correct” flag to run the read mapping bias correction algorithm, and the “multi-read-correct” flag to deal more appropriately with reads mapping to multiple locations. Finally, biological replicates were treated as such in the Cuffdiff analysis to account for this source of sample-to-sample variation. All downstream manipulation of the Cuffdiff output files was carried out in R (R Development Core Team 2011; http://www.R-project.org/) or Microsoft Excel. For each genotypic comparison, an FDR-adjusted *P*-value was generated for each gene model that accounted for multiple-testing [49]. Genes with corrected Q-values below 0.05 were considered significant in this analysis. The Cuffdiff files generated for pairwise genotypic comparisons are Additional files 1, 2, 3, 4 and 5.

### Annotation of predicted gene function

Genes were placed into seven categories based upon their Wormbase annotation. Transmembrane: predicted transmembrane domains annotated as “tmhmm”. Secreted: predicted signal peptide annotated as “SignalP”. Cytoplasmic cytoskeleton: predicted cytoskeletal protein or predicted interaction with the cytoskeleton (actin, microtubules, and intermediate filaments). Cytoplasmic signaling: predicted cytoplasmic molecules with known signaling functions, such as kinases, phosphatases, and ubiquitination. Cytoplasmic unsimilar: predicted cytoplasmic proteins with no known or predicted functions. Cytoplasmic metabolism: cytoplasmic proteins with predicted catalytic or biosynthetic activity. Transcription and gene expression: molecules predicted to be associated with transcription, splicing, or chromatin.

Gene ontology (GO) analysis was carried out using the Database for Annotation, Visualization and Integrated Discovery (DAVID: http://david.abcc.ncifcrf.gov/) [50,51]. The gene lists in Additional file 8 were subjected to analyses to discover enriched GO terms as well as functional clustering of GO terms. The enrichment lists and functional clustering outputs for each gene class can be found in Additional file 9.

### RNA-mediated gene interference (RNAi)

RNAi by feeding was conducted by standard techniques. RNAi clones were retrieved from an RNAi feeding library [36] (Source BioScience, Nottingham, UK). Only genes with representative clones in this library were tested (see Additional file 10). Each gene was tested twice independently on separate days. The data in Tables 6 and 7 represent pooled data from two independent tests. For each RNAi experiment, wild-type *mab-5* (*lqIs80 IV; lqIs58)* and *mab-5gof* (*mab-5(e1751) III; lqIs80 IV; lqIs58 V)* were grown on RNAi bacteria to assess effects in both wild-type and *mab-5gof* backgrounds. Furthermore, for each independent set of RNAi experiments (i.e. RNAi induction and bacterial growth), *ceh-20(RNAi)* was included as a positive control to ensure the efficacy of RNAi in both wild-type and *mab-5gof* backgrounds.

### Scoring AQR position

To score the final position of the QR descendant AQR, the position along the length of the animal was divided int five quadrants as described previously (Figure 2D): position 1 is the normal AQR position in the anterior deirid ganglion in the head; position 2 is posterior to the normal position but anterior to the vulva; position three is proximal to the vulva both anteriorly and posteriorly; position 4 is the normal birth position of QR near the posterior deirid; and position 5 is the posterior to the anus near the normal position of the QL descendant PQR in the phasmid ganglion. Differences between genotypes were determined by the percent of AQR cells in position 5 using Fisher’s Exact Test, and genotypes showing p < 0.05 significance level compared to *mab-5(e1751)gof* alone are shown in Tables 6 and 7.

## Competing interests

The authors declare that there are no competing interests.

## Authors’ contributions

JVT conducted RNAi experiments and commented on the manuscript; MG conducted genetic analyses and RNAi experiments and commented on the manuscript; SJM conducted computational analysis of the RNA seq data, wrote sections of the manuscript, and commented on the manuscript; EAL conducted RNA isolation, some computational analysis, some genetic and RNAi analysis, and wrote the manuscript. All authors read and approved the final manuscript.

## Supplementary Material

Additional file 1 **A Cuffdiff output file of gene expression differences between wild-type and *mab-5(e1239)lof.*** In all Cuffdiff output files, letters in the sample_1 and sample_2 columns represent the following: A is wild-type, B is *mab-5(e1239)lof*, C is *mab-5(gk670)gof*, and D is *mab-5(e1751)gof*. Click here for file

Additional file 2 **A Cuffdiff output file of gene expression differences between wild-type and *mab-5(gk670)lof.*** Click here for file

Additional file 3 **A Cuffdiff output file of gene expression differences between wild-type and *mab-5(e1751)gof.*** Click here for file

Additional file 4 **A Cuffdiff output file of gene expression differences between *mab-5(e1239)lof *and *mab-5(e1751)gof.*** Click here for file

Additional file 5 **A Cuffdiff output file of gene expression differences between *mab-5(gk670)lof *and *mab-5(e1751)gof.*** Click here for file

Additional file 6 **Genes with significantly different expression from Cuffdiff files in Additional files **1**, **2**, **3**,** 4 **and **5**. **Genes also found to be ChIP seq targets in Additional file 5 in [22] are indicated with a “Yes”. In all Cuffdiff output files, letters in the_1 and sample_2 columns represent the following: A is wild-type, B is *mab-5(e1239)lof*, C is *mab-5(gk670)gof*, and D is *mab-5(e1751)gof*. Click here for file

Additional file 7 Non-coding RNA genes that are differentially expressed in this RNA-seq analysis. Click here for file

Additional file 8 **Gene classes based upon functional annotation in Wormbase (see **Methods**).** Click here for file

Additional file 9 Output files from DAVID showing significantly enriched Gene Ontology terms and functional clusters involving genes identified by RNA-seq. Click here for file

Additional file 10 **Summary of RNAi results. **Genes that were upregulated in *mab-5(e1751)gof*, downregulated in *mab-5lof*, and were the top 50 predicted ChIP seq targets from Additional file 5 of [22] are listed. Indicated is whether or not the gene was tested by RNAi and whether or not RNAi modified posterior AQR migration in *mab-5(e1751)gof.* Genes included in each group are indicated by “also in *e1751* up*”*. Click here for file

Additional file 11 **Comparisons involving the known *mab-5* target gene *mig-13*.** In all Cuffdiff output files, letters in the sample_1 and sample_2 columns represent the following: A is wild-type, B is *mab-5(e1239)lof*, C is *mab-5(gk670)gof*, and D is *mab-5(e1751)gof*. Click here for file
